# Case report: Whole exome sequencing identifies a novel variant in the *HPRT1* gene in a male with developmental delay

**DOI:** 10.3389/fgene.2025.1512070

**Published:** 2025-02-28

**Authors:** Haoyang Zheng, Gui Chen, Tingting Wang, Weisheng Cheng, Jing Yuan, Fang Liu, Yuanhong Xu

**Affiliations:** ^1^ Department of Laboratory Medicine, The First Affiliated Hospital of Anhui Medical University, Hefei, China; ^2^ Anhui Key Laboratory of Infectious Diseases of Animal Origin, Anhui Medical University, Hefei, China; ^3^ Anhui Key Laboratory of Zoonoses, Anhui Medical University, Hefei, China; ^4^ Huangling Town Health Center, Fuyang, China; ^5^ Prenatal Diagnostic Center, Department of Obstetrics and Gynecology, The First Affiliated Hospital of Anhui Medical University, Hefei, China; ^6^ NHC Key Laboratory of Study on Abnormal Gametes and Reproductive Tract, Anhui Medical University, Hefei, China; ^7^ Engineering Research Center of Biopreservation and Artificial Organs, Ministry of Education, Hefei, China; ^8^ MOE Key Laboratory of Population Health Across Life Cycle, Hefei, China; ^9^ Anhui Province Key Laboratory of Reproductive Disorders and Obstetrics and Gynecology Diseases, Anhui Medical University, Hefei, China; ^10^ Anhui Provincial Institute of Translational Medicine, Anhui Medical University, Hefei, China

**Keywords:** lesch-nyhan syndrome, HPRT1, X-linked disease, HGPRT, novel variant

## Abstract

Lesch-Nyhan syndrome (LNS, OMIM #300322) is a rare X-linked genetic disorder caused by variants in the *HPRT1* gene, which codes for the Hypoxanthine-guanine phosphoribosyltransferase (HGPRT). *HPRT1* gene variants disrupt normal purine metabolism, leading to the involvement of multiple organ systems, primarily characterized by hyperuricemia, dystonia, and neurological abnormalities, which makes LNS clinically heterogeneous and diagnostically challenging. Here, we report a rare case of a 27-year-old Chinese male exhibiting severe lower limb motor disorders, hyperuricemia, and intellectual development delay. Blood tests showed hyperuricemia and whole exome sequencing (WES) identified a novel hemizygous variant in the *HPRT1* (NM-000194.3) gene: c.104T > C in exon 2, respectively. Bioinformatics techniques indicated that the variant may disrupt the activity of HGPRT. According to the clinical presentation, diagnostic examination, and WES results, the patient was finally diagnosed with LNS. This study identified a previously unreported pathogenic variant in the *HPRT1* gene. Although no curative therapy is currently available for *HPRT1* gene variants at present, a definite diagnosis of its genetic etiology is of great significance for genetic counseling and family planning.

## 1 Introduction

Lesch-Nyhan syndrome (LNS, OMIM #300322), also termed “self-destructive features syndrome,” is an autosomal recessive metabolic disorder. The typical clinical manifestations include self-injurious behavior (SIB), hyperuricemia, and a constellation of developmental delays, intellectual disability, and hypotonia. The incidence of this disease in Canada is estimated at 1/380,000, while in Spain it is approximately 1/250,000. However, it is less well-reported worldwide (Harris, 2018; Nanagiri and Shabbir, 2025). The causative gene *HPRT1* encodes hypoxanthine-guanine phosphor ribosyl transferase (HGPRT), which plays a central role in the remedial synthesis pathway of purine nucleotides by catalyzing the conversion of hypoxanthine and guanine to hypoxanthine nucleotides (IMP) and guanine nucleotides (GMP) (Bell et al., 2021).

A novel variant in the *HPRT1* gene (NM_000194.3: c.104T > C [p.Val35Ala]) was identified in this study by whole exome sequencing. The variant affects the function of HGPRT to some extent, and the patient exhibits typical LNS symptoms. LNS patients are less commonly reported in China (Chen et al., 2014; Huang et al., 2018; Jian et al., 2013; Li et al., 2022; Liu et al., 2015; Tong et al., 2022; Wang et al., 2023; Yang and Guo, 2022). Our research broadened the genetic and phenotypic diversity of LNS patients in China, while also offering important insights for genetic counseling.

## 2 Article types

This case report presents a Chinese patient with Lesch-Nyhan syndrome (LNS) who harbors a novel variant in the *HPRT1* gene.

## 3 Manuscript formatting

### 3.1 Materials and methods

#### 3.1.1 Ethics statement

The patient with Lesch-Nyhan syndrome were recruited from the First Affiliated Hospital of Anhui Medical University. This study received approval from the Ethics Committee of the First Affiliated Hospital of Anhui Medical University. Informed consent was procured from the patient and his family in consonance with institutional regulations and the Declaration of Helsinki. The patient and his family members received comprehensive clinical assessments performed by skilled doctors and laboratory staff at our hospital, and they submitted blood samples for genetic testing.

#### 3.1.2 Case presentation

The patient was a 27-year-old male with “congenital weakness of both lower limbs and inability to walk”. At the age of six, he could ambulate with the assistance of supportive devices. However, he subsequently experienced a gradual decline in his ability to walk, ultimately becoming wheelchair dependent. The patient did not show obvious self-injurious behavior during the clinical visit, but it was learned from the family that the patient is prone to emotional outbursts and may bang into walls. Additionally, the patient presented with characteristic features of developmental delay, cognitive impairment, and multiple gouty tophi. Laboratory tests showed that the concentration of uric acid in the patient’s blood was significantly higher than the normal level (Table 1).

**TABLE 1:** Biochemical examination indicators of the patient and his father.

	Urea (mmol/L)	Cre (μmol/L)	UA (μmol/L)
II3	5.36	89.0	847
I1	6.42	74.0	360

**FIGURE 1:**
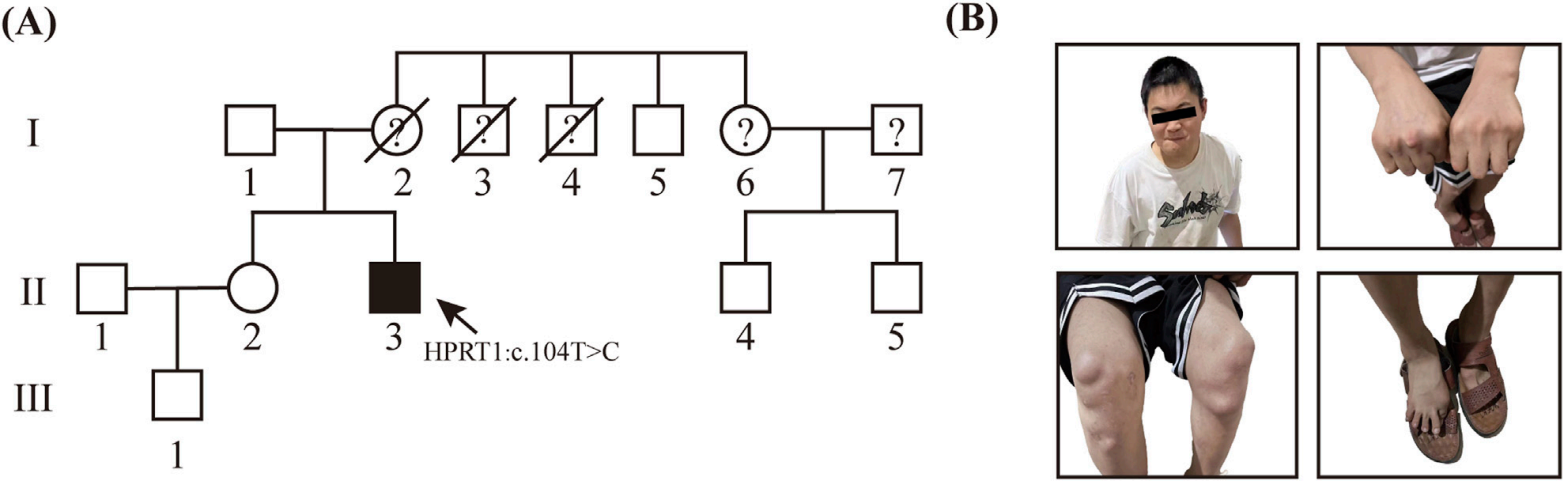
Genetic and Clinical Analysis of LNS in Unrelated Family **(A)** Precedenters’ family lineage diagram, which illustrates the inheritance pattern of LNS. **(B)** Hyperuricemia resulted in the occurrence of multiple gouty tophi in this patient.

At the time of the patient’s hospital admission, the patient’s mother was deceased. The patient’s father and sister were normal, and the parents were not consanguineous. Of the three uncles of the patient, one was phenotypically normal, while the remaining two displayed clinical symptoms comparable to those observed in the patient but have since passed away. Additionally, the patient’s cousin exhibited no phenotypical abnormalities and the patient’s mother did not present with similar symptoms during her lifetime. Due to the passage of time, the photographs and clinical records of the patient’s mother and two affected uncles were not adequately preserved, thereby limiting our ability to further corroborate the association between this variant and the disease. However, based on the available information, we suspect a maternal family history of Lesch-Nyhan syndrome. (Figure 1).

#### 3.1.3 Whole exome sequencing and variant calling

The information pertaining to the primary case and his family members was obtained from the complete autopsy report. DNA extraction was performed using the TIANamp DNA Extraction Kit (TIANGEN Biotech, DP348). DNA from the patient and their family members was extracted and quantified with a NanoDrop 3,000 spectrophotometer (Allsheng, Hangzhou, China).

Enriched DNA samples were sequenced on the AmCareSeq-2000 Sequencer (AmCare Genomics Lab, Ltd., Guangzhou, China). The sequencing targeted 20,324 genes associated with clinical manifestations in pre-certified individuals, covering a total of 209,175 coding regions. The average depth of coverage of the assay was 315 ± 146×, with 98.5% of the coverage intervals having an average depth of >10× and 98.4% of the coverage intervals having an average depth of >20×.

#### 3.1.4 Variant validation and sanger sequencing

The results of WES are analyzed and interpreted using a range of tools, including pathogenic variant databases such as ClinVar (https://www.ncbi.nlm.nih.gov/clinvar/) and HGMD (https://www.hgmd.cf.ac.uk/ac/index.php), databases of the normal population such as gnomAD (https://gnomad.broadinstitute.org/) and AllofUs (https://databrowser.researchallofus.org/), the Mendelian genetic disease database OMIM (https://www.omim.org/), and protein function prediction software PolyPhen-2 (http://genetics.bwh.harvard.edu/pph2/) and SIFT (https://sift.bii.a-star.edu.sg/). Concurrently, the genetic variants were screened and graded within the context of the test in accordance with the American College of Medical Genetics and Genomics (ACMG) variant classification guidelines and supplementary guidelines (Richards et al., 2015). Furthermore, the clinical manifestations of the patient and the corresponding test results were considered in order to identify rare variants that may be associated with the patient’s clinical manifestations. Subsequently, Sanger sequencing was conducted on DNA samples from the patient and his family members to confirm the pathogenic variants.

#### 3.1.5 Prediction of mutant protein structure

The amino acid sequence of the HGPRT protein was obtained from Uniprot (https://www.uniprot.org/). The 3D structures of both the wild-type and missense mutant were modeled using SwissModel (https://swissmodel.expasy.org/). Available crystal structures of HGPRT from the RCSB Protein Data Bank (http://www.rcsb.org) were used to refine the model. The protein’s functional domains were analyzed using the InterPro database (https://www.ebi.ac.uk/interpro/). Structures were visualized and analyzed with PyMOL.

### 3.2 Results

#### 3.2.1 Identification of hemizygous missense variants in the *HPRT1* gene

**FIGURE 2:**
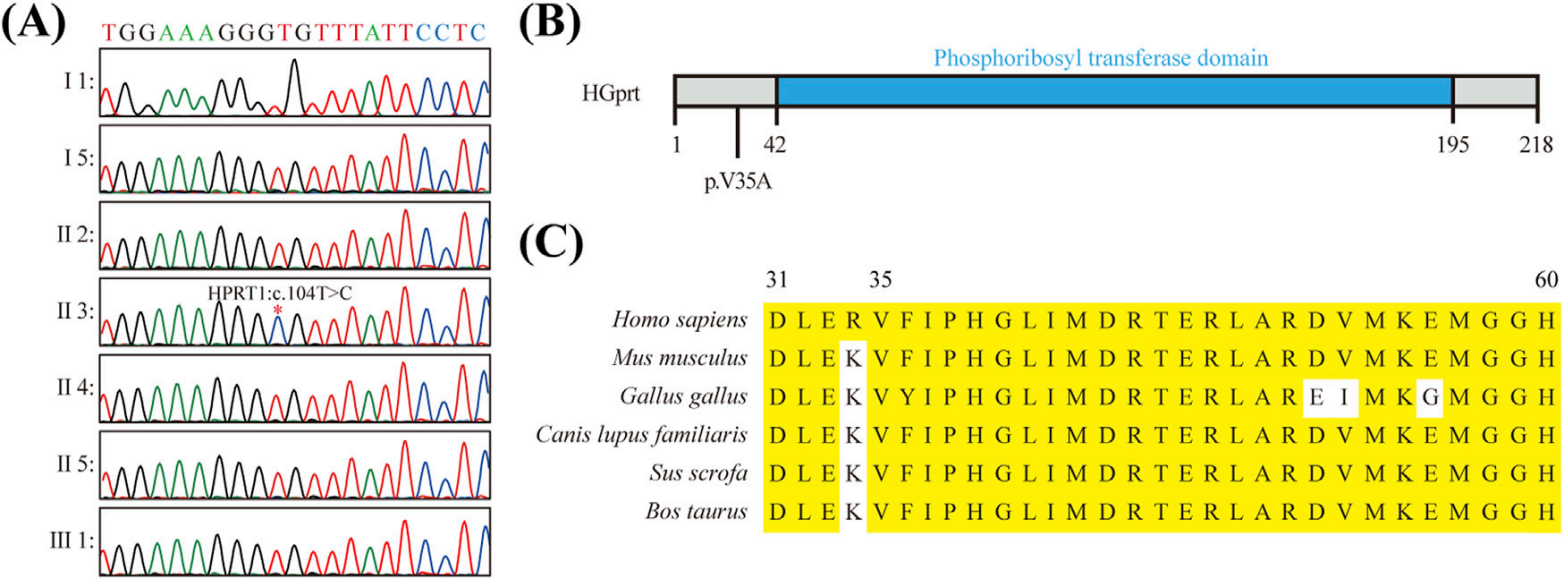
Molecular Characterization of *HPRT1* Missense Mutation **(A)** Sanger sequencing results verified the presence of the c.104T > C mutation in the HPRT1 gene of the preterist. **(B)** Schematic representation of the structural domain of the HGPRT protein and the position of the missense mutation c.104T > C (p.Val35Ala) within the nonstructural domain. **(C)** Amino acid sequence conservation analysis of the p.V35A mutation site in the HGPRT protein. The following amino acid sequence comparison results illustrate the high conservation of the valine residue at position 35.

To elucidate the pathogenic variants in this patient, a whole exome sequencing (WES) study of the patient’s DNA was performed. The genome of the patient was evaluated for single nucleotide variants (SNVs) and copy number variants (CNVs) in accordance with the guidelines established by the American College of Medical Genetics and Genomics (ACMG). It is regrettable to report that no disease-causing or potentially disease-causing variants were identified. Subsequently, all variants of unknown significance (VUS) were subjected to further analysis, which revealed a hemizygous missense variant in exon two of the *HPRT1* gene on the X chromosome (NM_000194.3: c.104T > C [p.Val35Ala]) (Supplementary Table S1). This finding was subsequently confirmed by Sanger sequencing. However, no relatives of the patient have been identified as carriers of this variant. When considered alongside the clinical manifestations observed in the patient’s family, the *HPRT1* c.104T > C variant probably originated from the patient’s mother (Figure 2A). Furthermore, this variant was not identified in various population-based databases, including the ClinVar, the All of Us Research Program and the Genome Aggregation Database, etc.

#### 3.2.2 Computational prediction of the pathogenic potential of the c.104T > C variant

To ascertain the suspected pathogenicity of the detected variant, a series of variant prediction tools were employed to analyze its potential harm. The results of the bioinformatics analysis indicated a high probability that the variant is indeed pathogenic. Moreover, this variant has not been identified in various population-based databases, including ClinVar, Genome AD (Genome Aggregation Database dataset), and AllofUs (All of Us Research Program). It is noteworthy that this variant is situated within a gene region that exhibits high levels of conservation (Figures 2B,C; Supplementary Table S2).

#### 3.2.3 Structural analysis of the mutated HGPRT protein

**FIGURE 3:**
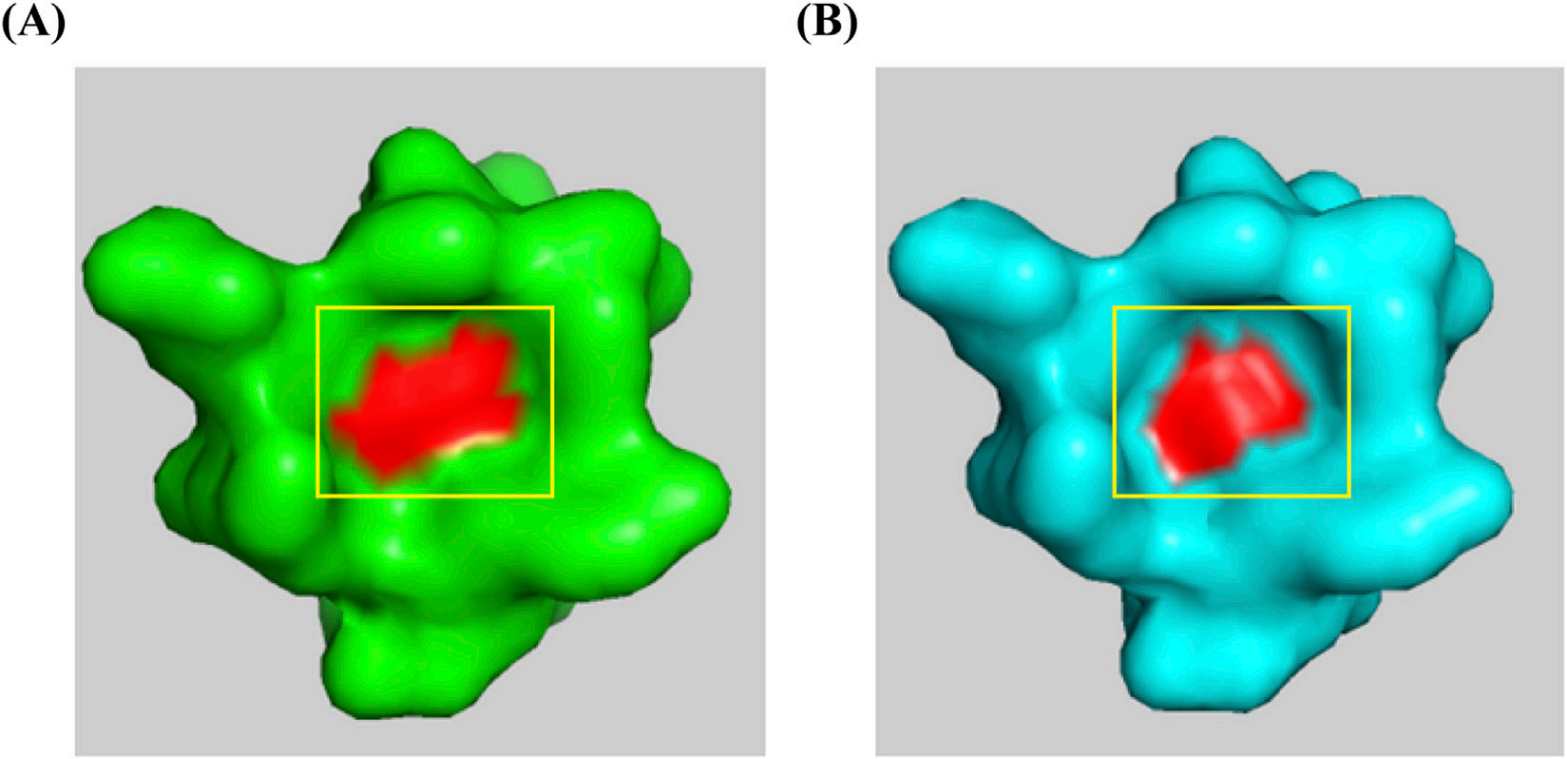
Structural comparison of wild-type and p.Val35Ala mutant HGPRT. The red area represents the 35th amino acid residue of the HGPRT protein. **(A)** Wild-type HGPRT structure showing the open concave pocket. **(B)** p.Val35Ala mutant HGPRT structure demonstrating the narrowing of the concave pocket due to the mutation at position 35.

The structural analysis of the p.Val35Ala mutation was conducted using SwissModel to predict the 3D structure of the HGprt protein, based on available crystal structures from the RCSB Protein Data Bank. Using InterPro to identify functional domains, we found that the mutation occurs within the Phosphoribosyltransferase domain (InterPro: IPR000836), a region located at the front of the catalytic site, potentially involved in substrate binding or enzyme activity regulation. Visualization using PyMOL revealed that the mutation causes a narrowing of a surface-exposed concave pocket, which is critical for ligand binding (Figure 3). This alteration in the pocket’s geometry could hinder substrate accessibility, thereby impairing the protein’s ability to bind its substrates. Based on this structural insight and the clinical manifestations observed in the patient, we hypothesize that this structural change may reduce the enzyme’s catalytic efficiency, supporting the pathogenicity of the p.Val35Ala variant.

### 3.3 Discussion

LNS is a rare disorder of metabolic abnormalities that was first described by Michael Lesch and William Nyhan in 1964 (Lesch and Nyhan, 1964). Patients with LNS typically present with an early onset of disease, often within the first year of age. The clinical manifestations of LNS are variable in their severity and may include hyperuricemia, movement disorders, intellectual disability, mental and behavioral abnormalities, such as recurrent self-injury and aggressive behavior. It is an X-linked recessive genetic disease. Reported patients are mainly male, and female patients are mostly carriers. In rare cases, female LNS patients have been reported (AlBakheet et al., 2022; Cho et al., 2019; Fu et al., 2014; Li et al., 2022; Tong et al., 2022). Early identification of hyperuricemia through blood tests is essential for the prognosis of LNS and highlights the importance of early diagnosis in rare disorders. Indeed, early diagnosis not only aids in symptom management but also plays a significant role in reducing long-term neurological damage. Importantly, genetic testing can provide critical information regarding inheritance patterns, offering families the opportunity to make informed decisions regarding reproductive planning.

The *HPRT1* gene is the causal gene for LNS, which is located in human Xq26.1 and has a total length of 40.5 kb. It consists of nine exons and eight introns and encodes 218 amino acids. To date, over 600 pathogenic variants have been documented, encompassing various forms of base substitutions, shifts, deletions, splices, and insertions (Nanagiri and Shabbir, 2025). Early genetic testing, particularly in families with a history of LNS, can help in understanding potential inheritance risks, reinforcing the need for genetic counseling and informed family planning.

The HGPRT protein, encoded by the *HPRT1* gene, is a ubiquitous cytoplasmic enzyme within the human body. Under normal physiological conditions, it catalyzes the conversion of hypoxanthine to hypoxanthine acid, guanine to guanosine-phosphate, and the metabolism of the purine moieties of hypoxanthine and guanine to uric acid (Fu et al., 2015). The synthesis of purine nucleotides in the human body occurs through two distinct pathways: the “*ab initio*” and the “remedial synthesis” pathways. However, the brain lacks the enzyme system necessary for the *ab initio* pathway, rendering it incapable of synthesizing purine nucleotides through this route. Consequently, HGPRT is expressed at a higher level in the brain, particularly in the basal ganglia, where the concentration of HGPRT is the highest. In patients with LNS, the reduced or absent activity of the HGPRT enzyme, which is encoded by the abnormal *HPRT1* gene, results in impaired purine metabolism in the body. This leads to excessive production of uric acid, which in turn causes hyperuricemia and related conditions such as gout and kidney stones (Guo et al., 2022). In addition, it has been found that HGPRT enzyme deficiency may affect the development of the dopamine system and cerebral cortex in the basal ganglia of the brain (Dinasarapu et al., 2022). The deposition of uric acid in the brain also results in the impairment of the basal ganglia, leading to cognitive deficits, neurological dysfunction, and SIB (Bell et al., 2021). The patient described exhibits significantly elevated serum uric acid levels, whereas his father’s uric acid levels are within the normal range. The patient presents with intellectual disability and severe motor impairments, characterized by congenital weakness in both lower limbs, preventing him from walking upright. Additionally, the patient demonstrates emotional instability, frequently engaging in self-injurious behaviors such as banging into walls. These metabolic disturbances highlight the importance of early diagnosis and genetic testing, which not only inform treatment decisions but also help identify at-risk family members. Genetic counseling can play an instrumental role in helping families understand inheritance patterns and potential risks.

The pathogenicity of abnormal *HPRT1* genes is mainly associated with reduced or absent HGPRT enzyme activity. HGPRT activity is mainly measured using whole blood, natural cells, solutions and other specimens to determine the HGPRT-catalyzed production of inosinic acid and guanosine from hypoxanthine and guanine, respectively (Lesch and Nyhan, 1964). LNS is classified into three types according to the degree of HGPRT activity impairment (Fu et al., 2014; Fu et al., 2015; Harris, 2018): (1) A complete lack of enzymatic activity (<1.5%) leads to classical LNS, with typical clinical features including hyperuricemia and its sequelae such as gouty arthritis and urinary tract stones, movement disorders such as dystonia, involuntary movements, cognitive impairment, and SIB; (2) HGPRT activity is partially lacking (1.5%–8%), resulting in a variant LNS, also known as an intermediate type, with relatively mild clinical symptoms; (3) The mildest phenotype is HPRT-related hyperuricemia (OMIM #300323), in which patients have HGPRT activity >8.0%. Some patients exhibit hyperuricemia and its accompanying symptoms, while symptoms of movement disorders and cognitive impairment are mild and can only be detected through a professional neurological examination (Keebaugh et al., 2007). Among them, the neurological function and behavioral abnormalities between Lesch-Nyhan syndrome and the HPRT-related hyperuricemia phenotype are referred to as HPRT-related neuro dysfunction. Patients have an HGPRT activity of 1.5%–8.0%. The clinical manifestations include hyperuricemia and neurological dysfunction, but no SIB (Fang et al., 2024; Guo et al., 2022; Jinnah, 1993). Through computer prediction and protein model construction of the *HPRT1*:p.Val35Ala variant carried by the patient in this study, we found that this variant resides within the phosphoribosyltransferase domain (IPR000836). This structural modification suggests that the variant may impair HGPRT enzyme activity by hindering substrate binding. These findings support the hypothesis that this variant is potentially deleterious. Based on the patient’s clinical manifestations, we speculate that this case represents a classic presentation of LNS. However, our hospital currently lacks the capability to perform HGPRT enzyme activity assays, which limits our ability to definitively confirm the diagnosis. This is a limitation of our study. We plan to conduct further research to validate these findings.

Compared with other reported *HPRT1* variants, the variant in our patient has its unique characteristics. For example, (Baba et al., 2017), reported a novel duplication variant (c.372_374 TTT > TTTT) in exon four of *HPRT1* in a 9-month-old boy. The variant led to abnormal splicing and manifested as developmental delay, athetosis, and dystonic postures without SIB at that time. In contrast, our patient, with a different variant type, not only had hyperuricemia and developmental delay but also more severe motor-function impairment, such as the complete inability to walk due to congenital lower - limb weakness (AlBakheet et al., 2022). described a novel frameshift variant in HPRT1 in a 4-year-old girl with LNS, presenting with global developmental delay, self-injurious behavior, hyperuricemia, hypotonia, speech delay, spasticity, and seizures. Our patient’s variant-induced symptoms showed differences in the frequency and intensity of self-injurious behavior and the pattern of motor-disorder manifestations. Zhang et al. (2019) reported two hotspot variants, c.508 C > T and c.151 C > T, which led to premature translation termination. These variants are different from the one in our patient at the genetic locus. Functionally, our patient’s variant had a more profound impact on the three-dimensional structure of the HGPRT protein, as predicted by *in silico* analysis, which might be related to the more complex clinical manifestations. Furthermore, we have conducted a comprehensive statistical analysis of LNS patients reported in China over the past 5 years (Supplementary Table S3).

Lesch-Nyhan syndrome is difficult to treat and mostly treated with symptomatic therapy. Pharmacological treatments mainly use allopurinol to control uric acid, while baclofen or benzodiazepines are used to treat muscle tone disorders (Qurie et al., 2025; Satow et al., 2021). Nevertheless, the optimal treatment for LNS’s motor and neurological symptoms remains under investigation. The most widely studied interventions are S-adenosylmethionine (SAM) and deep brain stimulation (DBS) (Benchoua et al., 2021; Deng et al., 2023), the former is considered an effective treatment for SIB in LNS patients (Krajewski et al., 2024). Botulinum toxin can also be used to treat dystonia in LNS; however, it is incapable of affecting other symptoms and can only be used as one of several adjuvant treatments for LNS (Dressler et al., 2021; Gilbert et al., 2021). Levodopa/carbidopa is reported to be beneficial for LNS treatment, early use of adequate doses of levodopa/carbidopa in LNS may prevent the occurrence of SIB (Visser et al., 2024). While these treatments provide symptom relief, they also raise important ethical considerations, especially regarding long-term use and the potential for quality-of-life impacts. Genetic counseling and informed decision-making become critical in such cases, ensuring that families can make the best choices regarding treatment options and future care. Additionally,to achieve precise correction of LNS, a previous study has utilized CRISPR-mediated cytosine base editors (CBEs) to introduce HPRT1 c.430C > T and c.508C > T variants in the HAP1 cell line, thereby establishing LNS disease models. Adenine base editors (ABEs) were subsequently employed to correct these variants, achieving repair efficiencies of 3% and 5.2%, respectively. Furthermore, prime editors (PEs) have been used for both constructing and repairing LNS disease models, with variant correction efficiencies reaching up to 14%. (Jang et al., 2023).

The potential of induced pluripotent stem cells (iPSCs) in modeling genetic diseases has opened new avenues for understanding disease mechanisms and developing novel treatments. iPSCs have been successfully applied to model a variety of genetic disorders, such as Rett’s syndrome, Angelman syndrome, Dravet syndrome, Cockayne’s syndrome and X-fragile syndrome (Camoes et al., 2023; Haase et al., 2021; Lee et al., 2022; Schuster et al., 2019; Szepanowski et al., 2024), providing valuable insights into disease pathogenesis and therapeutic interventions. By generating patient-specific models, iPSC technology enables drug screening and personalized therapy testing. In the context of Lesch-Nyhan syndrome, iPSC-derived neural models, including neural organoids, could serve as innovative platforms for studying the effects of HPRT1 variants and evaluating potential therapies. These models, previously used for neurological disorders like Parkinson’s disease (PD) (Kim et al., 2024), could be adapted to explore the impact of HGPRT deficiency on neural development and function, while high-throughput screening in these systems could help identify novel drugs to alleviate the neurological symptoms of LNS. A total of six potential therapeutic drugs have been evaluated using iPSCs derived from LNS patients (Ruillier et al., 2020). The drugs, including SAM, all contain at least one adenosine structure, and SAM has been tested in LNS patients. Meanwhile, studies have shown that chromosome transplantation (CT) in iPSCs derived from LNS patients may represent a potential therapeutic approach for LNS (Paulis et al., 2020).In addition, the experimental models should be further optimized with the use of neural organoids cultured from iPSCs, which would enable both a high throughput drug screening and investigation of disease mechanisms (Vandana et al., 2023).

Lesch-Nyhan syndrome is clinically heterogeneous, presenting with a range of manifestations including motor retardation, extrapyramidal and pyramidal tract symptoms. It is frequently misdiagnosed as cerebral palsy. The early detection of hyperuricemia through blood uric acid testing is crucial for the prognosis of Lesch-Nyhan syndrome. Early diagnosis and appropriate treatment can significantly improve the prognosis of Lesch-Nyhan syndrome. Genetic diagnosis is the key to preventing and controlling Lesch-Nyhan syndrome. Carrier screening and prenatal diagnosis can reduce the recurrence of Lesch-Nyhan syndrome in families.

### 3.4 Conclusion

This study identifies a novel variant in the *HPRT1* gene (*HPRT1*:c.104C > T) associated with Lesch-Nyhan Syndrome (LNS), a rare genetic disorder that leads to severe neurological symptoms. Our findings could improve the early diagnosis and genetic counseling for families affected by LNS, providing crucial insights for better management and prevention strategies.

## Data Availability

The original contributions presented in the study are included in the article/Supplementary Material, further inquiries can be directed to the corresponding author.
