# Supplementary material for: Case report: Whole exome sequencing identifies a novel variant in the HPRT1 gene in a male with developmental delay
Source: Front Genet. 2025 Feb 28;16:1512070. doi: 10.3389/fgene.2025.1512070 (PMC11906436; doi:10.3389/fgene.2025.1512070)
Supplement: Supplementary file 1 [file Table1.docx]

Supplementary Material

# Supplementary Figures and Tables

For more information on Supplementary Material and for details on the different file types accepted, please see [here](https://www.frontiersin.org/guidelines/author-guidelines#supplementary-material).

**Supplementary Table 1 Genetic test results and ACMG guideline ratings**

|  | cDNA variant: NM-000194.3 | Amino acid variant | Type of mutation | | ACMG classification | ACMG pathogenicity basis |
| --- | --- | --- | --- | --- | --- | --- |
| II2 | c.104T>C | p.V35A | Missense mutation | Hemizygous mutation | Uncertain significance (VUS) | PM2-P+PP3 |

**Supplementary Table 2 In silico analysis of HPRT1 variant (c.104T>C)**

| Tool | Score | Result |
| --- | --- | --- |
| SIFT | 0.02 | Deleterious |
| Polyphen-2 | 0.994 | Probably Damaging |
| FATHMM | -0.713 | Damaging |
| MutPred2 | 0.851 | Probably Damaging |

**Supplementary Table 3 HPRT1 Variants and Clinical Manifestations in Chinese Lesch-Nyhan Syndrome Patients Over the Past Five Years**

| Variants of *HPRT1* | Baseline hypotonia | Twisting | Spasms | Epilepsy | Developmental delay | Self-injurious behavior | Hyperuricemia | Study |
| --- | --- | --- | --- | --- | --- | --- | --- | --- |
| c.212_c.213insG | + | + | + | + | + | + | + | ([Li et al.,2022](#_nebED4BFD34_2F4D_430F_86F1_63BA258F690B)) |
| c.151C>T | + | + | + | - | + | + | + |  |
| c.299T>A | + | + | + | - | + | + | + |  |
| c.277_281delATTGC | + | + | + | - | + | + | + |  |
| loss | + | - | + | - | + | + | - |  |
| c.468_470delGAT | + | + | + | - | + | + | - |  |
| c.508C>T | + | - | + | - | + | + | + |  |
| c.500_501delGGinsC | All male individuals in this family exhibited classic symptoms of LNS and succumbed to the condition during childhood. | | | | | | | ([Tong et al.,2022](#_neb65942F7D_B4BA_42B6_A182_8A7956FE25F3)) |
| c.151C > T | + | + | - | - | + | - | + | ([Guo et al.,2022](#_nebA987ACD6_1AB0_4950_AA5F_E2443FD3E2F8)) |
| c.385-1G>C | + | + | + | - | + | + | + | ([Wang et al.,2023](#_neb970DE580_7CCB_490D_8E0B_7C7C8DD551DD)) |
| c.533-1G>C | + | + | - | - | + | - | + | ([Fang et al.,2024](#_nebBFB07AAE_D543_4D22_984A_6E5DB15E13DB)) |
| c.212dupG | + | + | + | + | + | - | + |  |

**References:**

Fang, H. H., Lee, C. L., Chen, H. J., Chuang, C. K., Chiu, H. C., and Chang, Y. H., et al. (2024). Whole exome sequencing facilitates early diagnosis of lesch-nyhan syndrome: a case series. [Case Reports; Journal Article]. *Diagnostics*. 14(24). doi: 10.3390/diagnostics14242809

Guo, M., Chen, Y., Lin, L., Wang, Y., Wang, A., and Yuan, F., et al. (2022). The study on the clinical phenotype and function of HPRT1 gene. [Journal Article]. *Child Neurol Open*. 9, 2329048X221108821. doi: 10.1177/2329048X221108821

Li, L., Qiao, X., Liu, F., Wang, J., Shen, H., and Fu, H., et al. (2022). Description of the molecular and phenotypic spectrum of lesch-nyhan disease in eight chinese patients. [Journal Article]. *Front. Genet.* 13, 868942. doi: 10.3389/fgene.2022.868942

Tong, M., Li, Q., Sun, A., Chen, C., Hu, S. (2022). [Analysis of HPRT1 gene variant and prenatal diagnosis for a chinese pedigree with lesch-nyhan syndrome but no specimen from affected probands]. [English Abstract; Journal Article]. *Zhonghua Yi Xue Yi Chuan Xue Za Zhi*. 39(11), 1243-1246. doi: 10.3760/cma.j.cn511374-20211029-00861

Wang, D., Zhao, J., Teng, J., Li, W., Zhao, X., and Li, L. (2023). [Genetic analysis of a chinese pedigree with lesch-nyhan syndrome]. [English Abstract; Journal Article]. *Zhonghua Yi Xue Yi Chuan Xue Za Zhi*. 40(6), 723-726. doi: 10.3760/cma.j.cn511374-20220916-00629
